# 

**Published:** 1905-11

**Authors:** 


Sixty-first Year.

Vol. LXI. NOVEMBER, 1905. No. 4

### BUFFALO MEDICAL JOURNAL

Established 1845 by AUSTIN FLINT, M. D.

NOV. 28. 190[illegible]

EDITOR:

WILLIAM WARREN POTTER, M. D.

ASSISTANT EDITORS:

WILLIAM C. KRAUSS, M. D.

NELSON W. WILSON, M. D.

ASSOCIATE EDITORS:
JAMES WRIGHT PUTNAM, M. D.
ERNEST WENDE, M. D.
JOHN PARMENTER, M. D.
JOHN A. MILLER, Ph. D.
HARVEY R. GAYLORD, M. D.
MAUD J. FRYE, M. D.

